# A New Calibration Method for Commercial RGB-D Sensors

**DOI:** 10.3390/s17061204

**Published:** 2017-05-24

**Authors:** Walid Darwish, Shenjun Tang, Wenbin Li, Wu Chen

**Affiliations:** 1 Department of Land Surveying & Geo-Informatics, The Hong Kong Polytechnic University, Hung Hom 999077, Hong Kong, China; w.darwish@connect.polyu.hk (W.D.); Shengjun.tang@whu.edu.cn (S.T.); wb.li@polyu.edu.hk (W.L.); 2 State Key Laboratory of Information Engineering in Surveying Mapping and Remote Sensing, Wuhan University, 129 Luoyu Road, Wuhan 430079, China; 3 Shenzhen Key Laboratory of Spatial Smart Sensing and Services & The Key Laboratory for Geo-Environment Monitoring of Coastal Zone of the National Administration of Surveying, Mapping and GeoInformation, Shenzhen University, Shenzhen 518060, China

**Keywords:** RGB-D sensors, depth calibration, structure sensor, structured light concept

## Abstract

Commercial RGB-D sensors such as Kinect and Structure Sensors have been widely used in the game industry, where geometric fidelity is not of utmost importance. For applications in which high quality 3D is required, i.e., 3D building models of centimeter-level accuracy, accurate and reliable calibrations of these sensors are required. This paper presents a new model for calibrating the depth measurements of RGB-D sensors based on the structured light concept. Additionally, a new automatic method is proposed for the calibration of all RGB-D parameters, including internal calibration parameters for all cameras, the baseline between the infrared and RGB cameras, and the depth error model. When compared with traditional calibration methods, this new model shows a significant improvement in depth precision for both near and far ranges.

## 1. Introduction

In recent years, RGB-D sensors have attracted great attention in 3D modeling due to their low cost. Two major concepts, time of flight (ToF) and structured light (SL), are used in RGB-D sensors. Many devices released in the market are based on these concepts; for example, Kinect v1, Structure Sensor [1] and ASUS Xtion Pro Live [2] are based on the SL concept, while Kinect v2 [3] is based on the ToF concept [4] The RGB-D sensor consists of three different sensors: an RGB camera, an infrared (IR) camera and an IR projector. In Kinect and ASUS, all three sensors are manufactured in a fixed frame, whereas the Structure Sensor combines two IR sensors only and is designed to be attached to a portable device with an RGB camera.

Although RGB-D sensors were originally designed to be used for gaming purposes such as remote controlling, they have recently made an important contribution to surveying and navigation applications, such as building information modeling, indoor navigation, and indoor 3D modeling [5,6] Although the accuracy required for gaming applications is not high, to extend the use of RGB-D sensors to surveying-type applications, accurate calibration of the device’s geometric parameters (i.e., camera focal lengths and baselines between cameras) and modeling the sensor errors (i.e., lens distortions and systematic depth errors) are necessary to produce high quality-spatial data and 3D models (e.g., cm-level precision).

The calibration of consumer-grade depth sensors has been widely investigated since the release of the first-generation Kinect in 2010. Various calibration methods, particularly for the depth sensor, have been studied by different research groups [6,7,8,9,10,11]. The calibration methods can be divided into four main categories.

The first category assumes that the three sensors (RGB camera, IR camera, and IR projector) behave similarly and pinhole camera concept is applicable to them all. This method is typified by [8] which uses the disparity data delivered from the IR camera and projector with the RGB image and a photogrammetric bundle adjustment to calibrate the internal and external parameters of the three sensors. Conventional distortion models [12,13] for each sensor separately compensate for their distortion effects. As it is difficult to obtain the raw measurements from the IR projector, the author computed such data from the disparity and the approximated baseline between the IR camera and projector. The main limitation of this category is the dependency between the observations from the IR camera and projector and their calibration parameters.

The second category combines disparity image produced by the depth sensor, the image produced by the RGB camera, and an empirical mathematical model to eliminate the distortions of the IR camera [9]. The distortion model is based on the error behavior of the whole RGB-D sensor. Unlike the first category, which is valid for any RGB-D sensor, this one is restricted to Kinect v1. Other limitations include the lack of automation and rigor. The user has to select the points in the depth image manually, and the homography matrix is computed using only four corresponding points.

The third category is dealing with the refinement of the in-factory calibration procedure, where the manufacture parameters including the baseline between IR camera and projector as well as the standard depth were precisely calibrated along with the RGB camera. Zhang’s [14] method is such a type of calibration; the author used the maximum likelihood estimation to calibrate color internal parameters and the manufacture parameters for a depth sensor without any distortion effect. The main limitation of this method is the distortion parameters for both IR cameras and projectors, which are not estimated or compensated.

Finally, the fourth category is mainly dealing with the depth calibration of the RGB-D sensor [15,16], the method adopts a mathematical error model derived from observation equation of depth measurements. The method used the output depth information from depth sensor and an adequate experiment setup to obtain the true depth of each pixel to model each pixel’s depth error. The method is valid for both SL and ToF RGB-D sensor. Although the error model was precisely applying to the middle part of the depth image, the whole image’s depth error model can be achieved and applied to the whole depth image. The error model has demonstrated a significant improvement while scanning small objects.

Based on these calibration algorithms, different calibration methods have been implemented and tested. Methods include the use of 1D [17] 2D [11], and 3D [18] calibration objects that work with the depth images directly; calibration of the manufacture parameters of the IR camera and projector [9,19]; or photogrammetric bundle adjustments used to model the systematic errors of the IR sensors [8,20]. To enhance the depth precision, additional depth error models are added to the calibration procedure [7,8,21,22,23]. All of these error models are used to compensate only for the distortion effect of the IR projector and camera. Other research works have been conducted to obtain the relative calibration between an RGB camera and an IR camera by accessing the IR camera [24,25,26]. This can achieve relatively high accuracy calibration parameters for a baseline between IR and RGB cameras, while the remaining limitation is that the distortion parameters for the IR camera cannot represent the full distortion effect for the depth sensor.

Two main issues faced by current RGB-D calibration procedures relate to the depth sensor. The first is the implementation of IR projector distortion along with the IR camera in the calibration procedure; the second is the correction of systematic errors resulting from the inaccuracy of in-factory calibration. This study addressed these issues using a two-step calibration procedure to calibrate all of the geometric parameters of RGB-D sensors. The first step was related to the joint calibration between the RGB and IR cameras, which was achieved by adopting the procedure discussed in [27] to compute the external baseline between the cameras and the distortion parameters of the RGB camera. The second step focused on the depth sensor calibration. First, the in-factory calibration parameters were updated to eliminate the systematic error resulting from the baseline between the IR camera and projector. Second, a combined distortion model was used to compensate for the distortion and systematic errors resulting from both the IR camera and projector. The experimental design and results are discussed in comparison with the conventional calibration method, and concluding remarks are made.

## 2. A Distortion Calibration Model for Depth Sensor

**Figure 1:**
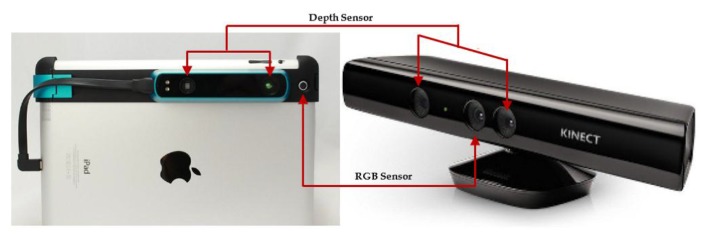
Main elements of RGB-D sensors (right: Microsoft Kinect [3]; left: Structure Sensor [1]).

Numerous RGB-D sensors were released on the market based on a structured light concept consisting of an IR camera and an IR projector. In addition to IR sensors, an optional RGB camera acquires the color information of the observed scene. The IR projector emits a predefined pattern and the IR camera receives it [28]. The depth of the image is obtained by triangulation based on the distance between their camera and projector. Figure 1 shows the main elements of the sensors, which use the SL concept. Two sensors are shown: Kinect v1 and the Structure Sensor. The main difference between both sensors is the baseline between the IR camera and projector. The longer the sensor’s baseline, the longer working distance can be achieved. The working range of Kinect v1 is 0.80 m to 4.0 m, while it is 0.35 m to 3.5 m for Structure Sensor.

**Figure 2:**
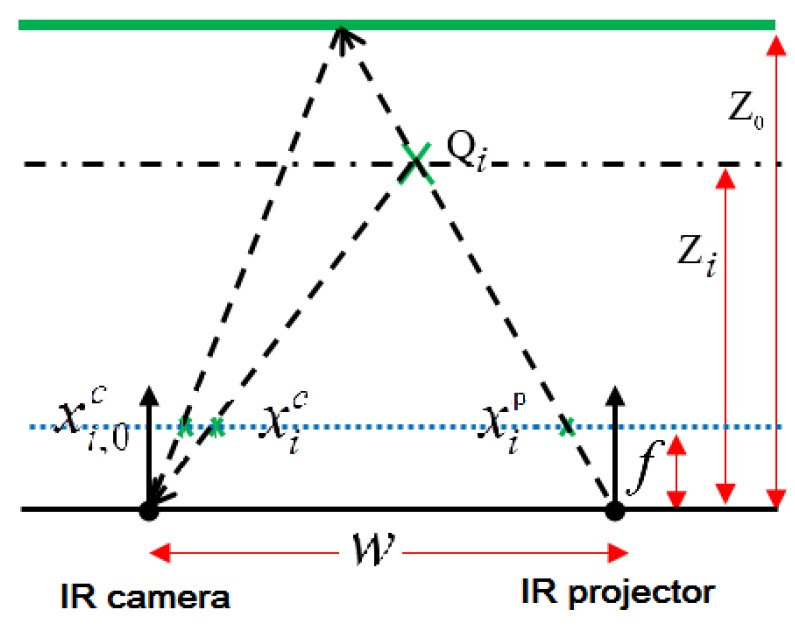
Depth computation concept from RGB-D sensors.

The principle of depth computation for RGB-D sensors is shown in Figure 2, where both IR sensors are integrated together to produce a pixel-shifted distance called disparity. The infrared projector pattern on a predefined plane (Z_0_) used in in-factory calibration [14] was stored in the sensor firmware. While capturing the real feature (*Q_i_*), both the IR standard projector pattern ($x_{i,0}^{c}$) and the real IR projector captured by IR camera ($x_{i}^{c}$) are identified. The difference between both locations is called disparity $d_{i}=x_{i}^{c}-x_{i,0}^{c}$. Using the disparity value and the predefined configuration information, including the focal length (*f*) of the IR sensors, the baseline between the IR projector and camera (w), and the standard depth of the projected pattern (Z0) [5,29], we can compute the depth of the feature point (*Q_i_* ).

Using the triangle similarity, the relationship between the standard pattern location ($x_{i,0}^{c}$), the real projector pattern location ($x_{i}^{c}$) in the IR camera space and the IR projector location ($x_{i}^{p}$) can be written as:

(1) $$x_{i,0}^{c}=x_{i}^{p}+fw/Z_{0}$$

(2) $$x_{i}^{c}=x_{i}^{p}+fw/Z_{i}$$

Applying the disparity $d_{i}=x_{i}^{c}-x_{i,0}^{c}$, the fundamental equation for computing the depth value for a feature point (*Q_i_* ) can be written as follows:

(3) $$Z_{i}=\frac{fw}{fw/Z_{0}+d_{i}}$$

As noted previously, the disparity value is measured by the firmware of the RGB-D sensor. However, the output value by the sensor is the normalized value ($d_{i}^{n}$), which ranges from 0 to 2047. The sensor uses (α) and (β) as two linear factors to normalize the measured disparity ($d_{i}$). The normalized disparity is stated as $d_{i}^{n}=\frac{1}{\alpha }\left(d_{i}-\beta \right)$. By substituting ($d_{i}^{n}$) in Equation (3) and combining all of the constants to assigned factors *a* and *b*, Equation (3) becomes:

(4) $$Z_{i}=\frac{1}{a+bd_{i}^{n}}$$

where a and *b* are constants:

(5) a=1Z0+βfwb=αfw

The final coordinates *X_i_*, *Y_i_*, and *Z_i_* for the acquired feature (*Q_i_*) are computed as:

(6) $$\begin{array}{l} Z_{i}=\frac{1}{a+bd_{i}^{n}}\\ X_{i}=\frac{x_{i}^{c}Z_{i}}{f}\\ Y_{i}=\frac{y_{i}^{c}Z_{i}}{f} \end{array}$$

The formula presented in Equation (4) with the factors *a* and *b* is called the manufacturer’s equation or the mapping function, which produces the depth information from the normalized disparity. The a and b coefficients are a function of the design parameters of the RGB-D sensor, which are the standard plane distance, the baseline, the focal length, and the linear parameters that convert the measured disparity to the normal disparity.

The baseline between IR camera and IR projector has a great influence in depth precision, using the covariance error propagation concept to estimate the variance of the depth resulting from SL RGB-D sensor. Using Equation (3) to estimate the depth variance, it can be figured out that $\sigma_{z}=z^{2}{\left(fw\right)}^{-1}\sigma_{d}$, where $\sigma_{z}$ and $\sigma_{d}$ are the precision of depth and disparity, respectively. For a certain SL RGB-D sensor, if the baseline (*w*) between IR camera and IR projector was increased to double, the precision of depth will be improved by 50% assuming all other variables were constants.

The disparity value can be computed from three constants, *f*, *w*, and Z_0_, and two measured quantities, $x_{i}^{c}$ and $x_{i}^{p}$. The measured quantities are affected by the distortion of the IR camera and projector, respectively. The systematic error can be assumed to be a function of the distortion parameters. The general expression that combines the effect of the systematic error and the distortion effect can be written as follows:

(7) $$d_{ti}=d_{i}+d_{e}$$

where $d_{ti}$ is the true disparity, $d_{i}$ is the measured disparity, and $d_{e}$ represents the error resulting from the effect of lens distortion and systematic error for the IR sensors.

The disparity error can be expressed as a function of the distortion effect for both the IR camera and projector for both tangential and radial distortions:

(8) $$d_{e}=\left(\delta_{\tan g.}^{c}+\delta_{rad.}^{c}\right)-\left(\delta_{\tan g.}^{c}+\delta_{rad.}^{c}\right)$$

where: $\delta_{\tan g.}^{c}$ and $\delta_{\tan g.}^{p}$ are the tangential distortion effect for the IR camera and projector, respectively, and $\delta_{rad.}^{c}$ and $\delta_{rad.}^{p}$ are the radial distortion effect for the IR camera and projector, respectively.

The radial distortion quantifies the lens quality, which is caused by bending the ray linked object, image, and focal points. The Brown model [30] with two factors (*K*_1_ and *K*_2_) is applied to compensate for the radial distortion. The tangential other model describes the distortion resulting from the inaccurate location of the lens with respect to the focal point, and the effect is compensated for using another two factors (*P*_1_ and *P*_2_) [12,31]:

(9) $$\begin{array}{l} \delta_{\tan g.}=P_{1}(({x_{t}}^{2}+{y^{2}}_{t})+2x_{t})+P_{2}x_{t}y_{t}\\ \delta_{rad.}=x_{t}(K_{1}({x_{t}}^{2}+{y^{2}}_{t})+K_{2}{\left({x_{t}}^{2}+{y^{2}}_{t}\right)}^{2}) \end{array}$$

where *P*_1_ and *P*_2_ are the factors represent the tangential distortion model, *K*_1_ and *K*_2_ are the factors represent the radial distortion model and $x_{t}$ and $y_{t}$ are the free distortion coordinates of the image point

As the disparity value is computed from the horizontal shift from the projected pattern to the standard one, the relation between the measured point coordinate and the true point coordinate can be expressed as follows:

(10) $$x_{m}=x_{t}+\delta_{\tan g.}+\delta_{rad.}$$

where $x_{m}$ is the measured x coordinate for the sensor and $x_{t}$ is the true x coordinate for the sensor.

Inserting Equation (9) into Equation (8), the full disparity error model considering both IR camera and projector distortions can be expressed in Equation (11) with eight parameters:

(11) $$\begin{array}{l} d_{e}={\left(P_{1}(({x_{t}}^{2}+{y^{2}}_{t})+2{x_{t}}^{2})+P_{2}x_{t}y_{t}\right)}_{c}-{\left(P_{1}(({x_{t}}^{2}+{y^{2}}_{t})+2{x_{t}}^{2})+P_{2}x_{t}y_{t}\right)}_{p}\\ +{\left(x_{t}(K_{1}({x_{t}}^{2}+{y^{2}}_{t})+K_{2}{\left({x_{t}}^{2}+{y^{2}}_{t}\right)}^{2})\right)}_{c}-{\left(x_{t}(K_{1}({x_{t}}^{2}+{y^{2}}_{t})+K_{2}{\left({x_{t}}^{2}+{y^{2}}_{t}\right)}^{2})\right)}_{p} \end{array}$$

where *p* refers to the IR projector and c refers to the IR camera.

To further simplify Equation (11), we used two parameters (*W*_1_ and *W*_2_) to describe the tangential distortion by correcting the relative orientation between the IR camera and projector lenses and applied another two parameters (*W*_3_ and *W*_4_) to describe the combined the radial distortion for both the IR camera and the projector, which can be considered as one lens combining the overlaying IR camera and projector lenses. As the relative orientation between the camera and projector is well fixed (as a rigid body) and pre-calibrated (mapping function calibration), the y axis for IR camera and IR projector can be assumed to be identical. The third and fourth terms of Equation (11) represent the radial distortion in the x direction resulting from the IR camera and projector. Due to the unknown projector data, we used the gross combined radial distortion, known as Seidal aberrations [12] and the IR camera’s pixel location to assign *x* distortion effect. This gives the two constraints shown in Equation (12):

(12) $$\begin{array}{l} y_{t}^{c}≅y_{t}^{p}\\ {\delta^{sensor}}_{rad.}=x_{t}^{c}F(x_{t}^{c},y_{t}^{c},W_{3},W_{4}) \end{array}$$

Equation (11) can then be simplified as:

(13) $$d_{e}=3W_{1}({x_{c}}^{2}-{x_{p}}^{2})+y_{c}W_{2}(x_{c}-x_{p})+W_{3}x_{c}({x_{c}}^{2}-{x_{p}}^{2})+W_{4}x_{c}({x_{c}}^{4}-{x_{p}}^{4}+2{y_{c}}^{2}({x_{c}}^{2}-{x_{p}}^{2}))$$

Equation (13) is the fundamental equation that describes the depth sensor distortion with only four parameters. As $x_{c}-x_{p}=d_{t}$ and $x_{c}+x_{p}=2x_{c}-d_{t}$, we have:

(14) $$\begin{array}{l} {x_{c}}^{2}-{x_{p}}^{2}=d_{t}(2x_{c}-d_{t})\\ {x_{c}}^{2}+{x_{p}}^{2}=d_{t}(2x_{c}-d_{t})+2{\left(x_{c}-d_{t}\right)}^{2} \end{array}$$

Finally, the full distortion model for the RGB-D sensor can be given as:

(15) $$d_{ei}={\left[\begin{array}{c} W_{1}\\ W_{2}\\ W_{3}\\ W_{4} \end{array}\right]}^{T}\left(\begin{array}{c} 3d_{ti}(2x_{i}-d_{ti})\\ 2y_{i}d_{ti}\\ x_{i}\left(d_{ti}(2x_{i}-d_{ti})\right)\\ x_{i}\left[\left(d_{ti}(2x_{i}-d_{ti})+2{\left(x_{i}-d_{ti}\right)}^{2})(d_{ti}(2x_{i}-d_{ti}))+2{y_{i}}^{2}d_{ti}(2x_{i}-d_{ti}\right)\right] \end{array}\right)$$

In Equation (15), the distortion model uses four parameters that consider both the radial and tangential distortions for both IR camera and projector lenses.

## 3. RGB-D Sensor Calibration

The calibration procedure was divided into two steps, with the first step handling the calibration of the RGB and IR cameras’ geometric parameters, including the focal length, principal point, and distortion parameters, and calibrated the RGB-IR baseline. The second step deals with the depth sensor calibration, whereby the manufacturer’s parameters and the proposed combined distortion model parameters are computed. In this section, we illustrate the methodology we used to achieve the two calibration steps.

### 3.1. RGB-D Joint Calibration

**Figure 3:**
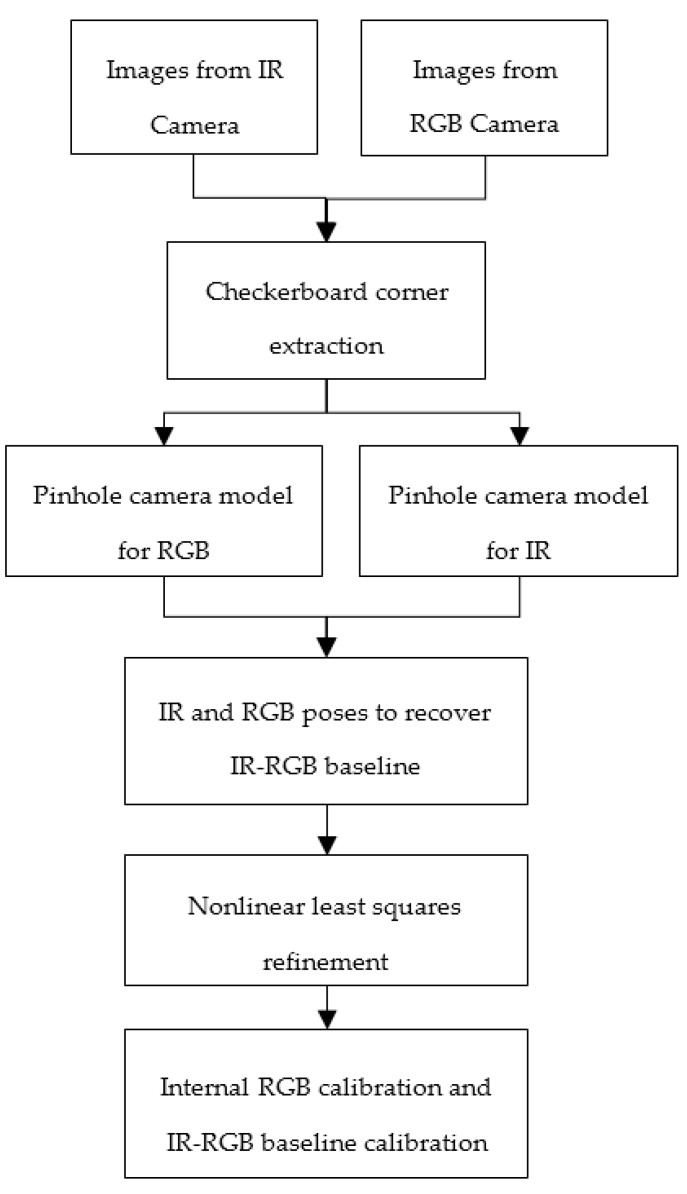
RGB-IR cameras baseline calibration methodology.

The methodology for jointly calibrating the RGB and IR cameras is indicated in the following chart (Figure 3). It started from stereo images produced by the RGB and IR cameras while the IR projector was switched off. After acquiring the stereo images, automatic corner extraction for a conventional A3 checkerboard was performed to extract the image points for each image. Using the ground coordinates of the checkerboard and extracted image points, a pinhole camera model was applied based on the calibration method discussed in [27] with five distortion parameters for each camera (three [*K*_1_ *K*_2_ *K*_3_] represented the tangential distortion and two [*P*_1_ *P*_2_] represented the radial distortion). After we optimized the external and internal parameters for both cameras, the global refinement was applied to enhance the estimated geometric parameters for each camera as well as the baseline between the RGB and IR cameras using Equation (16) [32]:

(16) $$\min\sum_{n}^{N}\sum_{m}^{M}\left({\left\|p_{mn}-\left(P_{mn}\left[\begin{array}{c} R_{n}\\ T_{n} \end{array}\right]\left[K\right]\right)\right\|}_{color}^{2}+{\left\|p_{mn}-\left(P_{mn}\left[\begin{array}{c} R_{n}\\ T_{n} \end{array}\right]\left[K\right]\right)\right\|}_{IR}^{2}\right)$$

where N is total number of images, M is the total number of points, $P_{mn}$ is the point pixel coordinates, $P_{mn}$ is the ground point coordinates, $R_{n}$ and $T_{n}$ are the rotation and translation matrix and K is the camera intrinsic matrix

### 3.2. Error and Distortion Model

**Figure 4:**
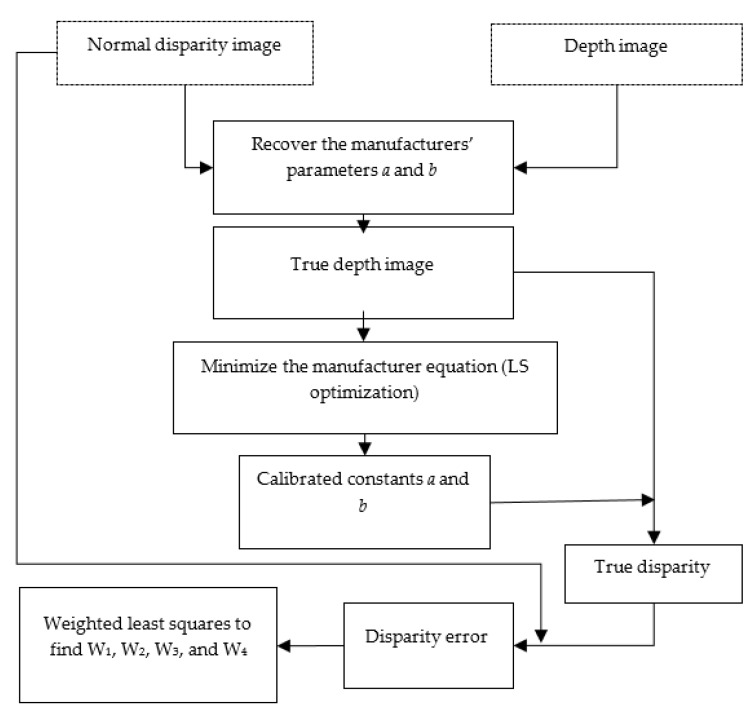
Computation methodology of depth sensor distortion model parameters.

The parameters calibrated in this step included the manufacturer constants *a* and *b* and the distortion parameters for depth sensors indicated in Equation (15). In this stage, the IR projector was switched on and the information produced by the depth sensor, presented in normal disparity and depth images, were acquired. After that, the recovery of manufacturer constants was estimated by applying Equation (4). Using the true depth and normal disparity, the manufacturer constants were calibrated using the least squares method, and the normal disparity was corrected based on calibrated *a* and *b*. The proposed distortion model was adopted to model the remaining distortion error resulted from the difference between true disparity and normal disparity. Using least square method with a weight matrix based on depth information, Equation (15) was solved and W’s parameters were computed. The methodology of the error model and distortion effect is introduced in Figure 4.

## 4. Experimental Design and Data Collection

Our experiments were designed to achieve the full calibration parameters for the RGB-D sensor. The calibration parameters were divided into three sets. The first set was the baseline between the RGB and IR cameras with the RGB camera’s internal parameters. The second set was the calibration of the mapping function (Equation (4)), which could consume the systematic error resulting from the baseline between the IR camera and projector, the standard depth, and the focal length of the depth sensor. The third set contained the distortion model parameters, which corrected the relative distortion resulting from the IR camera and projector. Dealing with the true value of observed plane, each sensor was attached to IPad device and setup on a movable tripod, a four control points were staked on each IPad. Using 0.50-m interval and starting from 0.50 to 3.00 m, six stations were identified. For each station, the control points in IPad were acquired using a total station, the distance between the sensor and observed plane was computed for the designated stations. Between the six stations, we collected several true depth images to enrich the observed data set. Based on the previously mentioned three steps, the data were collected in two phases. In the first phase, we collected a stereo-pairs image of the RGB and IR cameras for an ordinary chessboard to achieve the first set of calibration parameters. In the second phase, we collected pairs of depth and disparity images for a planar surface that we used to calibrate the depth sensor for the calibration parameters of sets two and three. We conducted the experiments on two different Structure Sensors. Table 1 shows the number of images collected to calibrate each sensor for two phases. Sensor 1 was attached to an iPad Air with serial number (S.N. 26779), while Sensor 2 was attached to an iPad Air 2 with serial number (S.N. 27414).

## 5. Calibration Results

The phase 1 data for each sensor were processed to compute the calibrated baseline between the RGB and IR cameras, while the phase 2 data were processed to calibrate the depth sensor. Table 2 and Table 3 are the results of phase1 for sensors 1 and 2. The output data are the internal parameters for the RGB and IR cameras. The internal parameters are represented as camera focal lengths (Fx, Fy) in pixels and principal point (Cx, Cy) in pixels and five distortion vectors (*K*_1_, *K*_2_, *P*_1_, *P*_2_, and *K*_3_), where K’s are introduced to consume the radial distortion effect and P’s are presented to eliminate the tangential distortion effect. The IR-RGB camera baseline is expressed in six parameters, including three translations (dx, dy, and dz) in mm and three rotation Euler angles (Rx, Ry, and Rz) in radians.

Table 4 shows the default parameters used by the firmware of the structure sensor, stated as the internal parameters for the depth sensor and color camera. The focal length and principal point for both sensors are the same, while the distortion parameters for both RGB and IR cameras are set are to zero.

After calibrating the baseline between the RGB and IR cameras and the internal parameters for the RGB camera, the phase 2 data were processed to calibrate the depth sensor. The two steps, including the mapping function calibration and distortion model, were conducted to deliver a high-precision depth measurement from the sensor. Table 5 shows the calibration result for mapping the function calibration. *a* and *b* are the mapping parameters mentioned in Equation (4).

**Figure 5:**
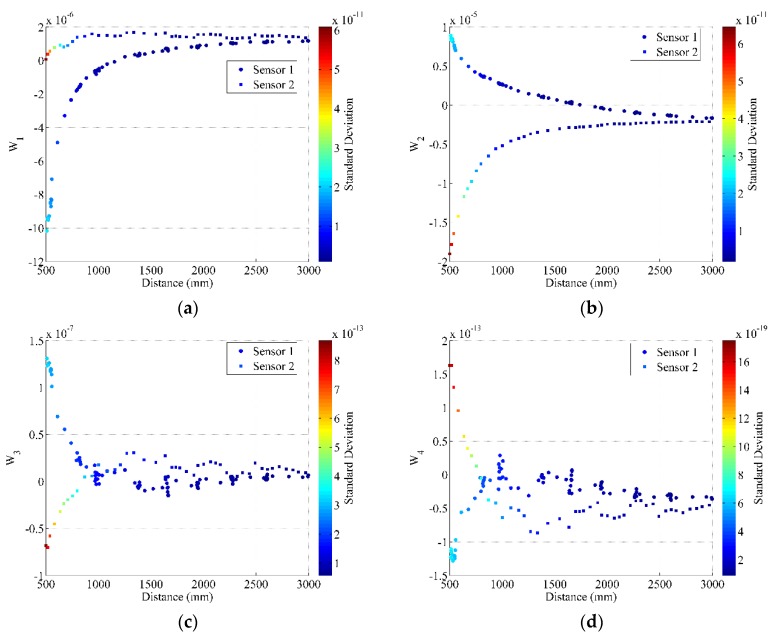
Distortion parameters for both sensors. (**a**) is *W*_1_; (**b**) is *W*_2_; (**c**) is *W*_3_; (**d**) is *W*_4_.

After computing the calibrated mapping function, we modified the measured depth and disparity information to correct the systematic error resulting from the mapping function error, then continued to compute the distortion model parameters. Figure 5 shows the distortion model parameters set for both Structure Sensors.

Figure 5 shows the main conclusion for the calibration procedure. Although the distortion parameters (*W*_1_, *W*_2_, *W*_3_, and *W*_4_) change with the measured distance, after 2.50 m, each distortion model parameter tends to be the same value. This means that for the full calibration parameters, it could be sufficient to collect the depth data with corresponding disparity images up to 2.50 m.

## 6. Accuracy Assessment of the Calibration Models

**Figure 6:**
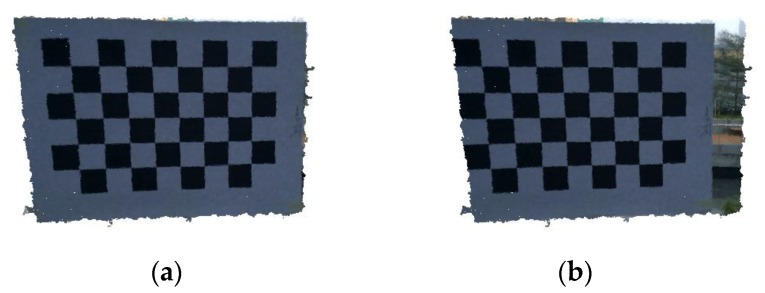
Alignment result for IR-RGB camera baseline (**a**) after applying calibration; (**b**) before applying calibration).

To examine our calibration methodology as well as the distortion model performance on the depth accuracy, we captured a new dataset for each sensor and applied the calibration results. To examine the IR-RGB baseline calibration, two images (depth and RGB) were collected and aligned using the calibrated parameters. Figure 6 shows the effect of the calibration parameters.

**Figure 7:**
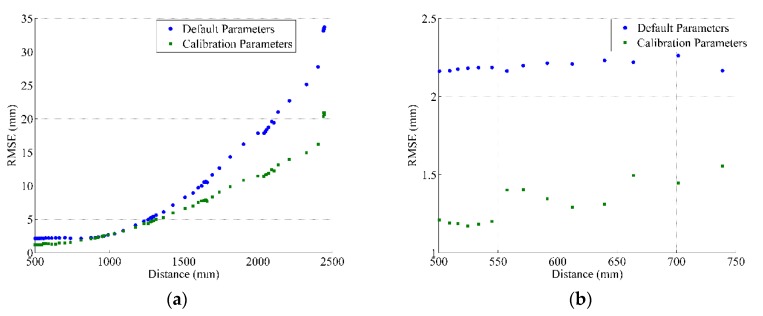
Depth precision variation with measured depth for Sensor 1. (**a**) the full range; (**b**) zoomed-in display of near range.

**Figure 8:**
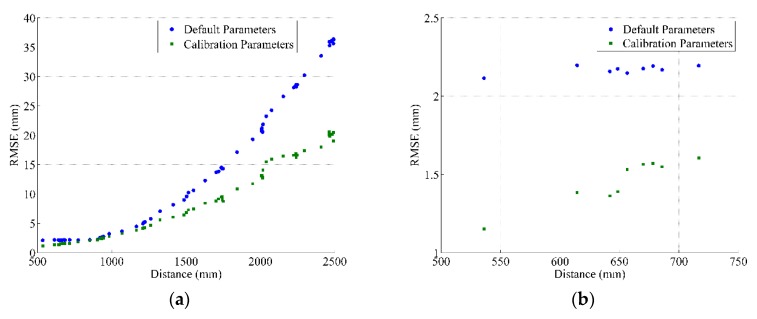
Depth precision variation with measured depth Sensor 2. (**a**) the full range; (**b**) zoomed-in display of near range.

To examine the performance of the depth calibration parameters, including the calibration of the mapping function and the distortion model, we collected several depth images for a planar surface and applied our calibration parameters. The examination criterion was based on the same procedure illustrated in [8,9]. Compared with the planes resulted from the total station, the RMSE of the fitted plane surface was used to describe the measured depth precision. Figure 7 and Figure 8 show the depth precision performance evaluation for sensors 1 and 2, respectively. The left side introduces the full range performance, and the right side zooms in to display the near range.

In addition to the planar surfaces assessment, two perpendicular planar surfaces (part of wall and ceiling) were captured using one of the calibrated sensors, the average distance between the sensor and the observed planes is 2.00 m (with minimum of 1.20 m and maximum of 3.00 m). The data were processed using the default parameters provided by the manufacturer and processed again after applying our distortion model. 

**Figure 9:**
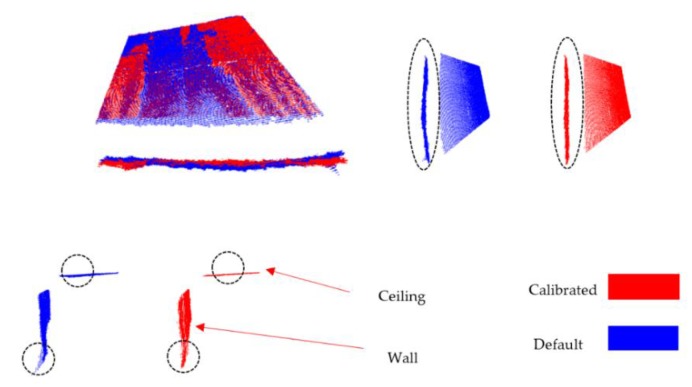
Point cloud of two perpendicular planes (blue color: default depth; red color: modeled depth): highlighted black dashed circles shows the significant impact of the calibration method on the point cloud quality.

Figure 9 shows the point cloud resulted from the RGB-D sensor before and after applying our distortion model. It is clearly seen that the warp in the wall was removed after calibration also the distortion on the depth image corners was significantly compensated. Comparison between the computed angle before and after applying our distortion model was shown in Table 6. Using different threshold for RANSAC to extract planes, the recovered average angle using our method is 89.897 ± 0.37 compared to 90.812 ± 7.17 using the default depth.

Comparing our results with those given in [9] our calibration method achieved nearly similar accuracy for the near range and a significant improvement in accuracy for the far range. However, our calibration method is simpler than the method given in [9] and the error model is based on a mathematical concept of the lens distortion effect.

## 7. Conclusions and Future Work

In this study, we propose a new method for calibrating the RGB-D sensor. This method can be applied to either three fixed cameras such as Kinect or separate systems like the Structure Sensor. The method was fully automated for both steps, which included calibrating the external RGB-IR baseline and modeling the distortion and depth error for a depth sensor. Based on the structured light concept, we also proposed a new distortion error model to compensate for the systematic and distortion effects for the RGB-D sensor. Better accuracy could be achieved for both the near and far ranges, compared with traditional calibration methods that used ordinary stereo calibration to produce a distortion model for a depth sensor or applied an empirical distortion model. Traditional calibration procedures can be used to achieve a significant improvement in depth accuracy for the near range (up to 50% accuracy improvement), which was already achieved in this procedure. For a far range, the traditional methods cannot be used to achieve any significant improvement in depth precision compared with our method; we could achieve an accuracy improvement of around 40% for the far range. The RGB-D sensor can extend up to 9.0 m in full depth range. Future research should focus on improving the depth uncertainty result from a small baseline between IR sensors to achieve a better depth precision for a far range. Calibration parameter stability over time and different light conditions must also be examined.

## Figures and Tables

**Table 1:** Data description for an RGB-D sensor (Structure Sensor).

Sensor	Phase 1		Phase 2	
	IR	RGB	Disparity	Depth
1	53		90	
2	59		44	

**Table 2:** The internal calibration parameters for both sensors.

Parameter		Fx (Pixels)	Fy (Pixels)	Cx (Pixels)	Cy (Pixels)	*K*_1_	*K*_2_	*K*_3_	*P*_1_	*P*_2_
**Sensor 1**	**RGB Camera**	592.59 ± 2.23	590.48 ± 2.04	305.69 ± 1.84	235.92 ± 1.30	0.019 ± 0.012	1.144 ± 0.091	−3.580 ± 0.291	−0.004 ± 0.001	−0.015 ± 0.002
	**IR Camera**	592.49 ± 2.17	591.22 ± 2.02	301.02 ± 1.76	239.9 ± 1.26	−0.13 ± 0.013	1.219 ± 0.131	−3.305 ± 0.476	0.000 ± 0.000	−0.014 ± 0.001
**Sensor 2**	**RGB Camera**	579.23 ± 2.22	580.29 ± 2.18	330.41 ± 1.43	242.63 ± 1.15	0.061 ± 0.014	0.744 ± 0.15	−3.607 ± 0.512	0.005 ± 0.001	0.006 ± 0.001
	**IR Camera**	568.57 ± 2.15	570.35 ± 2.11	323.83 ± 1.37	247.41 ± 1.16	−0.05 ± 0.018	0.343 ± 0.248	−0.982 ± 0.993	0.003 ± 0.001	0.003 ± 0.001

**Table 3:** The baseline calibration parameters for both sensors.

Parameter	Sensor 1	Sensor 2
**dx (mm)**	−37.997 ± 0.154	−32.603 ± 0.101
**dy (mm)**	−3.861 ± 0.117	0.431 ± 0.091
**dz (mm)**	−23.170 ± 0.656	−22.479 ± 0.473
**Rx (rad)**	0.0079 ± 0.0018	0.0110 ± 0.0021
**Ry (rad)**	−0.0024 ± 0.0025	−0.0044 ± 0.0028
**Rz (rad)**	−0.0053 ± 0.0002	0.0013 ± 0.0003

**Table 4:** The preset calibration parameters for the Structure Sensor.

Parameter	RGB Camera	IR Camera
**Fx (pixels)**	566.80	566.80
**Fy (pixels)**	566.80	566.80
**Cx (pixels)**	320	320
**Cy (pixels)**	240	240

**Table 5:** *a* and *b* values before and after calibration for both sensors

Sensor	In-Factory Calibrated Value		Calibrated Value	
	*a*	*b*	*a*	*b*
**1**	−3.38807 × 10^−6^	3.82665 × 10^−3^	−3.42936 × 10^−6^	3.86688 × 10^−3^
**2**	−3.38649 × 10^−6^	3.82538 × 10^−3^	−3.34912 × 10^−6^	3.78253 × 10^−3^

**Table 6:** Recovered angle between two perpendicular planes using our calibration method and default calibration.

RANSAC Threshold (m)	Recovered Angle (Degrees)	
	Default Depth	Modeled Depth
0.001	79.8288	89.8004
0.002	99.8740	89.3294
0.005	91.5966	89.9098
0.010	92.2871	90.2850
0.020	90.4728	90.1596
